# Sjögren‐Larsson syndrome: The mild end of the phenotypic spectrum

**DOI:** 10.1002/jmd2.12099

**Published:** 2020-03-25

**Authors:** Pippa Staps, Judith van Gaalen, Peter. van Domburg, Peter M. Steijlen, Sacha Ferdinandusse, Tom den Heijer, Marieke M. B. Seyger, Thomas Theelen, Michèl A. A. P. Willemsen

**Affiliations:** ^1^ Department of Pediatric Neurology Radboud University Medical Center, Amalia Children's Hospital, Donders Institute for Brain Cognition and Behaviour Nijmegen The Netherlands; ^2^ Department of Neurology, Donders Institute for Brain Cognition and Behaviour Radboud University Medical Center Nijmegen The Netherlands; ^3^ Department of Neurology Zuyderland Medical Center Sittard The Netherlands; ^4^ Department of Dermatology, The GROW School for Oncology and Developmental Biology Maastricht University Medical Center Maastricht The Netherlands; ^5^ Laboratory Genetic Metabolic Diseases, Amsterdam Gastroenterology & Metabolism Amsterdam UMC, University of Amsterdam Amsterdam The Netherlands; ^6^ Department of Neurology Franciscus Gasthuis and Vlietland Rotterdam The Netherlands; ^7^ Department of Dermatology Radboud University Medical Center Nijmegen The Netherlands; ^8^ Department of Ophthalmology, Donders Institute for Brain Cognition and Behaviour Radboud University Medical Center Nijmegen The Netherlands

**Keywords:** ALDH3A2, FALDH, Sjögren‐Larsson syndrome, spastic diplegia

## Abstract

Sjögren‐Larsson syndrome (SLS) is a rare inborn error of lipid metabolism. The syndrome is caused by mutations in the *ALDH3A2* gene, resulting in a deficiency of fatty aldehyde dehydrogenase. Most patients have a clearly recognizable severe phenotype, with congenital ichthyosis, intellectual disability, and spastic diplegia. In this study, we describe two patients with a remarkably mild phenotype. In both patients, males with actual ages of 45 and 61 years, the diagnosis was only established at an adult age. Their skin had been moderately affected from childhood onward, and both men remained ambulant with mild spasticity of their legs. Cognitive development, as reflected by school performance and professional career, had been unremarkable. Magnetic resonance spectroscopy of the first patient was lacking the characteristic lipid peak. We performed a literature search to identify additional SLS patients with a mild phenotype. We compared the clinical, radiologic, and molecular features of the mildly affected patients with the classical phenotype. We found 10 cases in the literature with a molecular proven diagnosis and a mild phenotype. Neither a genotype‐phenotype correlation nor an alternative explanation for the strikingly mild phenotypes was found. New biochemical techniques to study the underlying metabolic defect in SLS, like lipidomics, may in the future help to unravel the reasons for the exceptionally mild phenotypes. In the meantime, it is important to recognize these mildly affected patients to provide them with appropriate care and genetic counseling, and to increase our insights in the true disease spectrum of SLS.

**SYNOPSIS**

Sjögren‐Larsson syndrome (OMIM #270200) is a recognizable clinical disorder that almost always leads to a (classic) severe phenotype; in this study, we learn that mild phenotypes exist, with the same genetic and biochemical abnormalities, and normal intelligence but otherwise essentially the same—but far less severe—neurocutaneous syndrome.

## 1 INTRODUCTION

In 1957, Sjögren and Larsson described a syndrome with congenital ichthyosis, intellectual disability, and spastic diplegia, in a series of 28 Swedish patients with a rather homogeneous clinical phenotype, enabling the recognition of the disorder that has since been called Sjögren‐Larsson syndrome (SLS).^1^

In 1988, it was found that abnormalities in the lipid metabolism were responsible for the clinical features of SLS.^2^ The enzyme involved, fatty aldehyde dehydrogenase (FALDH), is part of the fatty alcohol nicotinamide adenine dinucleotide oxidoreductase complex and catalyses oxidation of many different medium‐ and long‐chain fatty aldehydes, derived from fatty alcohols, into fatty acids. FALDH deficiency results in the accumulation of fatty aldehydes and fatty alcohols, which is considered the principal causative disease mechanism in SLS. In 1996, the *ALDH3A2* gene was discovered to be coding for the FALDH enzyme. *ALDH3A2* mutations have been identified in all patients with SLS.^3^ To date, more than 100 mutations in the *ALDH3A2* gene have been described,^4^ and all patients, except some extremely rare cases, appear to suffer from essentially the same, aforementioned clinical triad of symptoms, with only minor variations in severity.^5^, ^6^, ^7^, ^8^, ^9^

Our group has several decades of experience with patient care for children and adults with SLS, as well as clinical SLS research. We have learned to know SLS in its common, severe form, and had—among more than 30 patients—recognized only one sib pair with a milder phenotype in the past (cases H1 and H2 of SLS^10^ and cases 18 and 19 of SLS^7^). Recently, a third patient with a remarkably mild SLS phenotype was referred to our clinic.

Here we describe two of the three mildly affected Dutch patients (one patient did not give informed consent). Additionally, we performed a literature study to identify other mild cases of SLS. We aim to increase awareness and expand the phenotypic spectrum of SLS. By making comparisons between the mild vs classical presentation, we hope to identify the factors involved in determining the clinical outcome and understand the natural history, thus improving our insights into the underlying disease mechanism.

## 2 METHODS

### 2.1 Patient studies

We performed an observational study on two unrelated, male, adult patients from the Netherlands, with a mild SLS phenotype. The study was performed according to the tenets of the declaration of Helsinki (2013 revision), and was approved by the Regional Committee on Research Involving Human Subjects. Informed consent was obtained from both participants prior to inclusion in the study.

The patients were seen in our outpatient clinic in 2018. Full physical and neurological examinations were performed. Additionally, a detailed ophthalmologic examination was performed to study visual function and retinal morphology. Visual acuity was measured using Snellen charts. Slit‐lamp examination, ophthalmoscopy, optical coherence tomography (OCT), and fundus photography were performed. In patient 1, cerebral magnetic resonance imaging (MRI) and MR‐spectroscopy (MRS) were performed. Patient 2 underwent MR imaging in 1995, but did not give consent for a follow‐up scan.

### 2.2 Literature study

We searched in PubMed for papers in which individual cases of SLS were described in sufficient detail. We only included patients with a biochemically or genetically proven diagnosis. We searched on PubMed using “Sjögren‐Larsson syndrome” as search term. All papers on SLS, published from 1988 (first report about the enzyme defect) to September 1, 2019 (date of searching), were screened for descriptions of patients with “nonclassical” phenotypes. Only English papers were included. Cases were defined as being nonclassical, that is, mild, when one of the core symptoms (ichthyosis, intellectual disability, and spasticity) was missing or when at least two of the three core symptoms were described as being mild.

## 3 RESULTS

### 3.1 Case descriptions

#### 3.1.1 Patient 1

**Figure 1:**
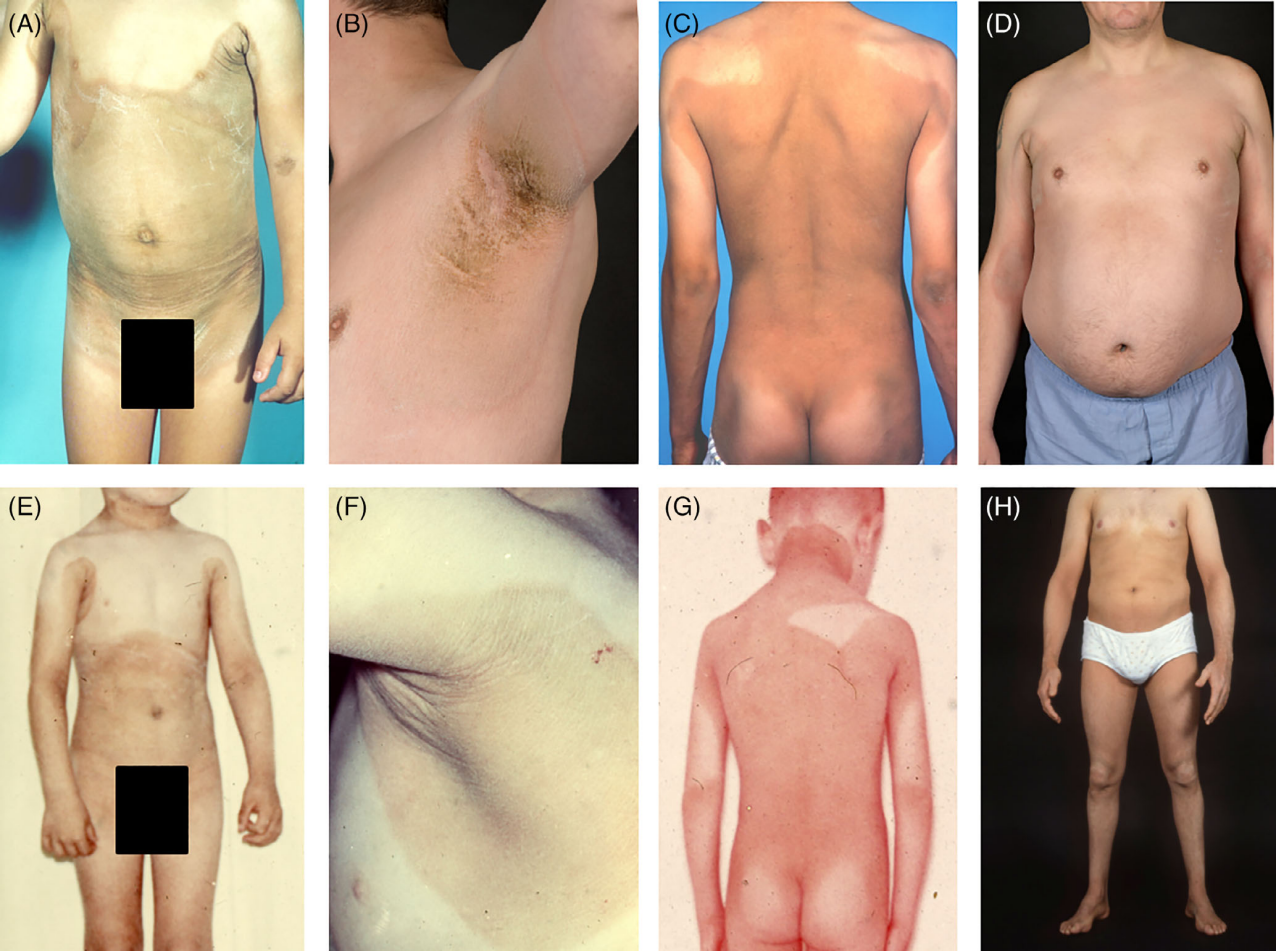
Skin abnormalities of patients 1 (A‐D) and 2 (E‐H). Skin photographs of patients 1 and 2 at different ages. A,E, Photography of the trunk of patient 1 at the age of 3 years (A) and of patient 2 at the age of 7 years (E). A sharply demarcated hyperpigmented, ichthyosiform plaque is seen. B,F, Close‐up photography of the right axilla of both patients. C,G, Photography of the back of both patients (C, patient 1 at the age of 17 years; G, patient 2 at the age of 7 years), with a sharply demarcated hyperpigmented ichthyosiform plaque. D,H, Photography of the trunk of patient 1 at the age of 45 years (D) and adult age of patient 2 (H). Sharply demarcated, partially hyperkeratotic plaques with lichenification and an ichthyosiform desquamation in the axillae and medial aspects of the arms and elbows are seen in patient 1. In patient 2, the abdomen, axillae, and medial aspects of the arms and elbows are affected and sharply demarcated

This 45‐year‐old male patient presented at our hospital with a new diagnosis of SLS. He was born late preterm, with a dry skin. In the following years, ichthyosis developed, covering mainly his trunk and showing sharply demarcated affected areas (Figure 1A‐C). From the age of 30 years on, his ichthyosis became less evident (Figure 1D), but he still suffered from considerable pruritus. During childhood and early adulthood, his motor skills had been considered “clumsy,” but the patient neither experience stiffness nor needed adjusted shoes. Neurological examination after experiencing a traffic accident at the age of 30 years showed—for the first time—a clear (but mild) spastic gait. He required glasses from already a young age and experienced photophobia. A formal IQ test had never been performed but based on his school carrier (he finished high school), employment history (he worked in a stockroom), and general performance, his cognitive functions were considered normal. His speech was unremarkable. His parents were nonconsanguineous, and he had one healthy brother. His spastic gait had been attributed to his preterm birth until a neurologist put all the symptoms together and tested the patient for SLS at the age of 45 years.

**Figure 2:**
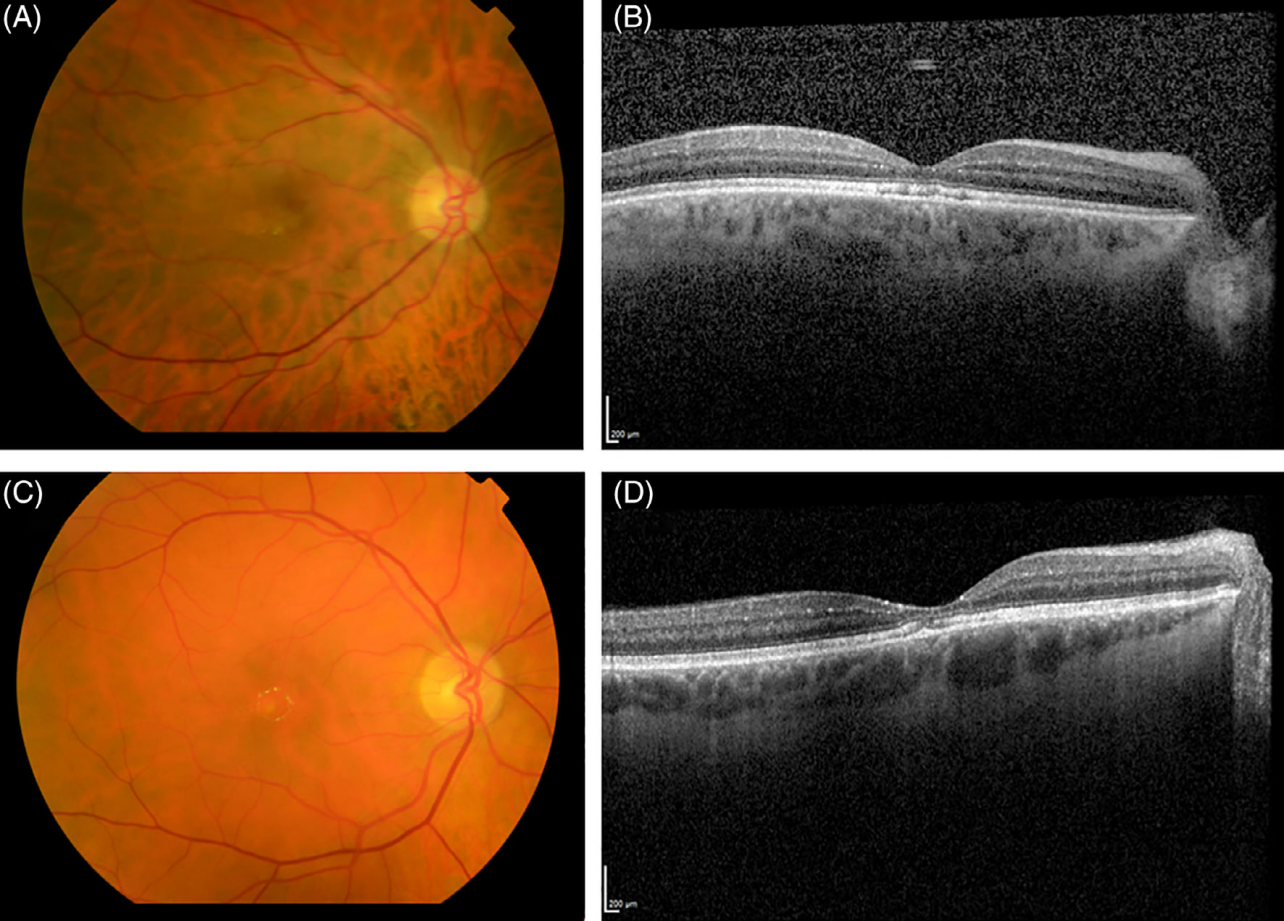
Retinal abnormalities of patients 1 and 2. A, Fundus photography of patient 1 at the age of 45 years, with a few perifoveal crystals visible around the macula lutea together with a lack of the expected physiological darkening. B, Macular OCT scan of patient 1, with a thinned macula, hyperreflective dots suggesting crystalline deposits and interruptions of the photoreceptor layer visible suitable with SLS maculopathy. Next to this, a mild central serous chorioretinopathy is seen. C, Fundus photography of patient 2 at the age of 61 years, with parafoveal crystals visible. D, Macular OCT scan of patient 2, with also a thinned macula and changes in the retinal pigment epithelium. OCT, optical coherence tomography

On examination, a good‐natured man was seen. In the axillae, groins, and the lumbar region, ichthyosis was present. Remarkably, the medial parts of the upper arms were affected, whereas the lateral parts were not (Figure 1B and D). On the legs a mild, diffuse ichthyosiform desquamation was seen; the face, neck, most of the trunk, and lateral sides of the arms were not affected. He showed a spastic paraplegia, but was able to walk without aids. Visual acuity was 0.5 and 0.4 for the right and left eye, respectively, and findings on ophthalmoscopy and OCT were compatible with SLS maculopathy (Figure 2 A and B).

**Figure 3:**
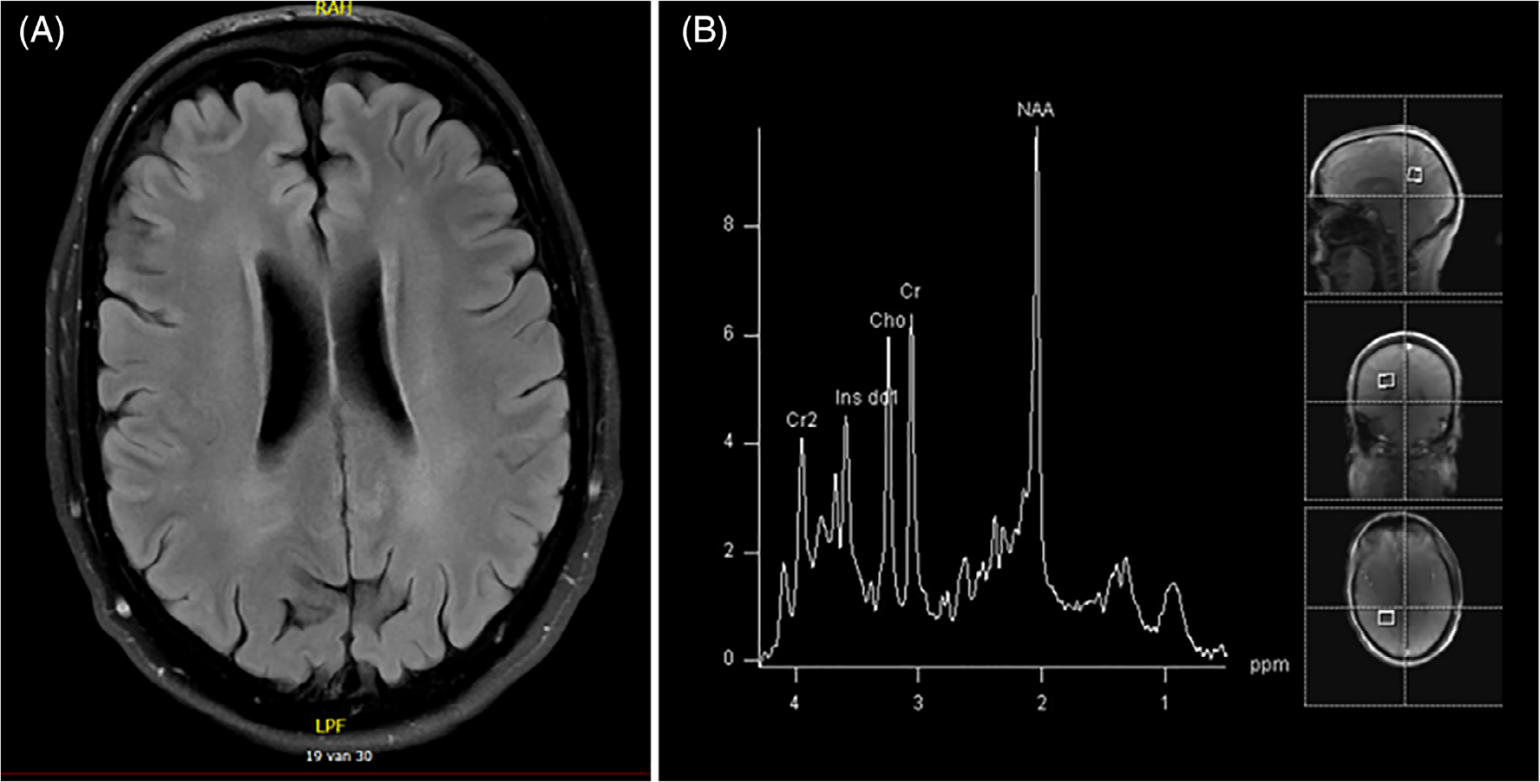
Cerebral magnetic resonance imaging and spectroscopy of patient 1. A; T2‐weightened MRI scan with subtle abnormalities in the periventricular white matter. B, MRS from the white matter, without the characteristic lipid peaks. MRI, magnetic resonance imaging; MRS, magnetic resonance spectroscopy

Cerebral MRI showed diffuse, subtle signal changes of the periventricular white matter without other abnormalities. MRS revealed normal spectra of the parieto‐occipital white and occipital gray matter, and (thus) did not show the typical “lipid peak” at 1.3 ppm (Figure 3).^11^ FALDH activity in lymphocytes was below detection limit of the enzyme assay. Sanger sequencing showed compound heterozygosity for two pathogenic variants in the *ALDH3A2* gene, namely, c.682C>T (p.[Arg228Cys]) and c.943C>T (p.[Pro315Ser]).

#### 3.1.2 Patient 2

This 61‐year‐old male patient was already known for more than 25 years in our clinic, and we have described his phenotype previously.^7^, ^10^, ^11^, ^12^ He was diagnosed with SLS at the age of 34 years after clinical suspicion, using a targeted enzyme analysis. He was born at term. He was noted to have dry skin at the age of approximately 9 months, later described to be lamellar ichthyosis. A sharp demarcation of the skin abnormalities was seen (Figure 1, E‐G). Despite the use of acitretin, his skin showed a subtle ichthyosiform desquamation which was still pruritic (Figure 1H). At the age of 33 years, a mild spastic paraplegia was diagnosed after adolescent‐onset spastic paraplegia. The patient was able to walk without assistance, with adjusted shoes. He suffered from a mild visual impairment and photophobia. He had a (low) normal intelligence with an IQ of 83 and a mild speech impairment. He was married and had two healthy children. He has one affected sister with SLS and five unaffected healthy siblings. The affected sister had an overall phenotype that was even slightly milder than the phenotype of her brother.^7^, ^10^

On examination, a pleasant, mildly dysarthric man was seen. Mild ichthyosis was seen on the back, in the axillae and the back of his legs. The remainder of the skin appeared normal. He showed a mild spastic diplegia, with mild contractures of the ankles. Tendon reflexes were increased in the legs and he had a bilateral Babinski sign. His arms were neurologically unaffected. Best corrected visual acuity was 0.4 for both eyes. On slit‐lamp exam, a bilateral cortical and nuclear cataract was seen. Ophthalmoscopy revealed crystalline deposits around the macula, and the physiological retinal darkening caused by macular pigment was lacking (Figure 2C). Retinal imaging using OCT showed a thinned macula, with hyperreflective dots and an interrupted photoreceptor layer in both eyes (Figure 2D). In the left eye, foveal microcysts were seen. At the age of 38 years, an MRI of the brain had been performed, which showed mild periventricular hyperintense signals and mild ventricular enlargement. No MRS was performed. Biochemical and molecular analysis previously had shown FALDH deficiency in fibroblasts, and compound heterozygosity for two pathogenic variants in the *ALDH3A2* gene, namely, c.551C>T (p.[Thr184Met]) and c.943C>T (p.[Pro315Ser]).

### 3.2 Case reports in literature

A review of literature, revealed six publications describing 10 non‐Dutch patients with a mild phenotype (Table 1).^13^, ^14^, ^15^, ^16^, ^17^, ^18^ Two additional case reports describing mildly affected patients were excluded due to insufficient biochemical or genetic information.^19^, ^20^

**Table 1:** Case reports found in the literature describing patients with a mild phenotype of Sjögren‐Larsson syndrome

Case number	Paper	Case in paper	Age	Sex	Skin	Eyes	Spasticity	Speech	Intellectual disability	Gestational age	FAO/FALDH activity	*ALDH3A2* mutation
1	Nigro^13^	A	14 y	F	Ichthyosis on abdomen, axillae, extremities. Less pronounced lesions on back and face	No abnormalities on ophthalmologic evaluations	Gradually worsened spasticity, however able to walk without assistance	Normal	No evidence of mental retardation, but diagnosed as having a learning disability	ND	6/not tested (pmol/min/mg protein; normal 75 ± 26)	ND
2		B	12 y	M	Ichthyosis on legs, axillae, soles	No abnormalities on ophthalmologic evaluations	Gradually worsened spasticity, however, able to walk without assistance	Normal	No evidence of mental retardation, but diagnosed as having a learning disability	ND	2/398 (pmol/min/mg protein; normal 75 ± 26/8540 ± 1158)	ND
3		C	7 y	F	No abnormalities	ND	Minimally abnormal gait	Normal	No evidence of mental retardation	ND	3/257 (pmol/min/mg protein; normal 75 ± 26/8540 ± 1158)	ND
4	Kawakami^14^	1	13 mo	M	Mild ichthyosis of lower abdomen and dorsal aspects of extremities	No abnormalities	No history of spastic diplegia, but he had club feet	ND	Developmental delay	Normal pregnancy and delivery	Not tested/694 ± 212 PMol/min/mg (mean ± SD; normal 2536 ± 649).	ND
5		2	5 y	M	Mild ichthyosis on his lower abdomen and the dorsal aspects of his extremities	No abnormalities	Walk without assistance, no history of spastic diplegia	Normal	Learning disability, mental retardation gradually worsened	ND	Histochemical testing revealed reduced alcohol dehydrogenase activity in the ichthyotic skin, similar to that found in patient 1 in this paper	ND
6	Carney^15^	1	4 y	ND	Ichthyosis+	ND	+/Ambulatory	ND	Mild mental retardation	ND	FALDH activity 9%	c.286_296del/c.1268G>A
7	Didona^16^	1	12 y	F	Ichthyosis became evident during the first month of life	Pigmentary retinopathy was ruled out	Due to spasticity in the legs, the patient first walked at 24 months of age only with support, on her tip‐toes, and with adducted hips and flexed knees. She underwent surgical correction of leg contractures and started to walk independently with a spastic gait at 7 years of age. Since then, her motor disability has remained stable and currently, at 12 years of age, spasticity involves only the lower limbs	Normal language development	Moderate learning difficulties	ND	Not tested	c.769insA (homozygous)
8		2	5 y	M	Ichthyosis present at birth	Pigmentary retinopathy was ruled out	Leg spasticity became evident in the subsequent few months, but the diplegia remained mild and surgical intervention has been avoided so far	ND	Mild mental retardation	ND	935 pmol/min/mg (normal values 6750‐20 570)	c.1094C>T, c.471+2T>G
9	Tachibana^17^	1	5 y	F	Ichthyosis on trunk, limbs	ND	Spasticity in both legs, MRI mild abnormalities, MRS lipid peak	ND	No deficiencies in intellectual development	39 wk	ND	c.1339A>G, c.504_505insAG
10	Papathemeli^18^	1	3 y	F	Ichthyosis on axillae, neck, palms, soles, flexures	BCVA 0.5/0.3, fundoscopy and OCT normal	Bilateral spasticity of legs. MRI white matter lesions, MRS moderate increase lipid	ND	Normal (IQ 95)	39 wk	ND	c.551C>T (homozygous)

Abbreviations: BCVA, best corrected visual acuity; F, female; M, male; ND, not described.

The case reports included children from ages 1 to 14. The cases described different spectrum of severity for the three cardinal clinical symptoms. In three patients, the ichthyosis was described as being absent or mild. In seven patients, the spasticity was mild, for example, reflected by the fact that these patients were ambulatory. Intellectual development was normal in eight patients. Considering other prevalent symptoms, ophthalmological abnormalities were not seen in six out of seven patients in whom data were reported, and speech was normal in five patients (out of the five in whom it was reported explicitly). Gestational age was only described in three patients; all three were born at term. All five patients in whom mutation analysis had been performed had different *ALDH3A2* gene mutations. In most of them, and in all remaining cases without a genetic diagnosis, enzyme deficiency had confirmed the clinical diagnosis of SLS.

## 4 DISCUSSION

We describe two adult patients with both biochemical and genetical confirmations of SLS, presenting with a milder spectrum of this disorder from the classical form. Both patients display a rather straight‐forward neurological disorder with normal cognitive functions, and adolescent‐ or adult‐onset spastic paraplegia with slow progression and preservation of ambulation during at least decades. Preterm birth is typically associated with the classical phenotype of SLS, and it has not been documented in mildly affected cases.^21^ The ichthyosis of patients 1 and 2 occurred early in life but did not follow the distinctive pattern seen in the classical phenotype. Interestingly, pruritus was a key complaint similar to the classical phenotype, and also normal appearing skin was felt to be itchy. Patients with “milder SLS phenotypes” thus appear to suffer from essentially the same clinical syndrome, although to a lesser degree. This is further confirmed by the observation that the retinal abnormalities of patients 1 and 2 were also similar to the classical SLS patients, showing a thinned retina with crystalloid depositions and interruptions of the photoreceptor layer.^22^

Applying MRS, lipid accumulation can be demonstrated in cerebral white matter of patients with classic SLS. Remarkably, the spectra of patient 1 did not show the typical resonances reflecting lipid accumulation. The other mildly affected Dutch patient known in our center, the sister of patient 2, did not demonstrate the typical lipid peak on MRS in previous studies.^7^, ^10^ The absence of lipid accumulation, or at least much lower brain tissue concentrations of abnormal lipids (below the detection limit of MRS), might be one of the explanations for the mild neurological phenotype.

The 10 clinical descriptions found in the literature of mildly affected patients with genetically or biochemically proven SLS are not uniform; the extent to which brain, skin, and eyes are affected varies between patients (Table 1). All patients were children. Patient 5 was only 13 months old at the time of reporting, thus may not express all the symptoms. It is well‐possible other adult patients with mild phenotype exist but are not diagnosed—and described in literature—yet.

We tried to understand why some patients show a milder phenotype, because this might contribute to a better understanding of the disease mechanisms underlying SLS. Enzyme activity measurements were not useful for this purpose because there is no clear correlation between residual enzyme activity and clinical phenotype.^23^ Because FALDH is a member of a large family of aldehyde dehydrogenases,^24^ most available enzyme assays are not 100% specific for FALDH activity. This hampers the ability to reliably determine small amounts of residual FALDH enzyme activity and consequently stands in the way of investigations studying a potential relationship between severity of the phenotype and residual FALDH activity.

We focused on the mutations of patients 1 and 2, and searched the mutations in Pubmed and the Leiden Open Variation Database (LOVD, https://databases.lovd.nl/shared/genes/ALDH3A2). The first variant in patient 1 (c.682C>T) was described in six siblings in a homozygous state and a variable phenotype.^5^ Although the patients in this cohort had slightly variable patterns of severity across symptoms, we think that they still display the clearly recognizable, common SLS phenotype. The second mutation of patient 1 (c.943C>T) is one of the most common mutations in the classical SLS population. Patient 2 has this same c.943C>T mutation. The second mutation of patient 2 (c.551C>T) is described in two other cases: the milder case from the report of Papathemeli et al^18^ (case 10 in Table 1) and a patient with a classical phenotype.^25^ The mutations described in the mild cases identified in our literature review were compared with the LOVD database as well and listed in Table 2. Altogether, we concluded that there is no clear genotype‐phenotype correlation to explain the mild phenotype of some patients with SLS. This lack of genotype‐phenotype correlation was also described in a recent paper by Abdel‐Hamid et al.^31^

**Table 2:** *ALDH3A2* gene mutations in mildly affected patients with Sjögren‐Larsson syndrome

Case^a^	Mutation	Other cases found in LOVD database		
		Papers	Clinical features	FALDH activity
NL1	c.682C>T	Lossos^5^	Variable patterns of severity in SLS symptoms	4% of normal
		Shamriz^26^	Classical SLS, coexistence with cytidine deaminase deficiency	Not described
		Hidalgo^27^	Classical SLS	Not described
NL1; NL2	c.943C>T	Willemsen^7^	Classical SLS	Reduced
		Ganemo^28^	Classical SLS	Not described
		Sillen^29^	Not described	Not described
		Rizzo^30^	Classical SLS	Reduced
NL2; 10	c.551C>T	Jean‐Francois^25^	Classical SLS	Not described
6	c.286_296del	No	Not applicable	Not applicable
	c.1268G>A	No	Not applicable	Not applicable
7	c.769insA	No	Not applicable	Not applicable
8	c.1094C>T	Sillen^29^	Not described	Not described
		Rizzo^30^	Classical SLS	Reduced
	c.471+2T>G	Rizzo^30^	Classical SLS	Reduced
9	c.1339A>G	No	Not applicable	Not applicable
	c.504_505insAG	No	Not applicable	Not applicable

Abbreviations: LOVD, Leiden Open Variation Database; SLS, Sjögren‐Larsson syndrome.

a Cases NL1 and NL2 are the described cases from the current paper; cases 7‐10 correspond with Table 1.

Apart from a (missing) genotype‐phenotype correlation, other mechanisms could potentially affect the severity of the phenotype in SLS. First, mildly affected patients may have “active alternative metabolic routes” to escape the metabolic defect, or may have “upstream” variants that decrease the flux of lipids through the pathways in which FALDH plays a role.

Second, mosaicism or revertant mosaicism of the genetic mutation might explain the peculiar distribution of the affected and nonaffected skin in patients 1 and 2.^32^, ^33^ However, the pattern of the ichthyosis in these patients (Figure 1) looks similar and does not fit any of the described mosaic distributions.^32^, ^34^, ^35^

Irrespective of the lack of understanding of the underlying mechanisms, we find it important to increase the awareness of the mild phenotype of SLS. Patients with this phenotype could present to neurologists, ophthalmologists, and dermatologists, and it is important to recognize this nonclassical phenotype of SLS. These patients should receive appropriate (genetic) counseling, and may possibly need different treatment approaches in the future. Studying these patients hopefully may increase our insights into the disease, both on an individual level (with a chance on better personalized care) and on a group level (with a more complete definition of the disease spectrum and better understanding of the underlying disease mechanisms).

## CONFLICT OF INTEREST

The authors declare no potential conflict of interest.

## AUTHOR CONTRIBUTIONS

P.S.: execution of the study, clinical assessment of patients, data interpretation, writing the manuscript. J.v.G.: clinical assessment of patients, correction of manuscript. P.v.D.: clinical assessment of patients, correction of manuscript. P.M.S.: clinical assessment of patients, correction of manuscript. S.F.: enzyme activity measurements, correction of manuscript. T.d.H.: correction of manuscript. M.M.B.S.: clinical assessment of patients, correction of manuscript. T.T.: clinical assessment of patients, correction of manuscript. M.A.A.P.W.: design of the study, clinical assessment of patients, data interpretation, correction of manuscript. M.A.A.P.W. is the guarantor for the article

## INFORMED CONSENT

All procedures followed were in accordance with the ethical standards of the responsible committee on human experimentation (institutional and national) and with the Helsinki Declaration of 1975, as revised in 2013. Informed consent was obtained from all patients for being included in the study.

## DATA AVAILABILITY

Patient data are saved in the electronic patient records of our hospital.
